# Meloxicam-Induced Pancreatitis

**DOI:** 10.7759/cureus.12976

**Published:** 2021-01-28

**Authors:** Eric Landa, Ismail Ganim, Erika Vigandt, Talhah Siraj, Ying Zhu

**Affiliations:** 1 Internal Medicine, Unity Health, Searcy, USA; 2 Internal Medicine, Brooklyn Hospital, Brooklyn, USA

**Keywords:** pancreas, pancreatitis, meloxicam, gastrointestinal, drug induced pancreatitis

## Abstract

There are 525 drugs that have been identified by the World Health Organization (WHO) as having the potential to cause pancreatitis. The most well-known drugs include mesalamine, azathioprine, and simvastatin, all of which have been well described in the literature. However, drug-induced pancreatitis only used to account for about 1%-2% of cases in the 1990s; this number has increased to up to 5% in some studies. By accounting for over 100,000 cases per year in the United States alone, it is important to be able to recognize these cases and act rapidly and appropriately to remove the offending agent. The vast majority of cases occur within six weeks of initiating or increasing the dosage of such medications. Here we present an interesting case of meloxicam-induced pancreatitis.

## Introduction

Pancreatitis is the leading cause of gastrointestinal-related hospitalizations in the United States. Although the etiology may vary slightly depending on what part of the world you find yourself in, the top three causes include gallstone induced, alcohol intake, and hypertriglyceridemia. Other less common but important etiologies include trauma, medication-induced, and medical procedures such as endoscopic retrograde cholangiopancreatography (ERCP). There are well-documented cases of medication-induced pancreatitis even though the mechanism of action by which this occurs is not fully understood by a majority of them. In addition, some of these medications have not been well documented. To the best of our knowledge, the number of meloxicam-induced pancreatitis reports is few. Herein we report such a case.

## Case presentation

A 59-year-old Caucasian male with a past medical history of diabetes mellitus type II presented to the emergency department complaining of abdominal pain. He reported the pain started three days prior after having finished eating his dinner; it started suddenly and was localized to his epigastrium with radiation to his back. He described the pain as sharp and constant, rated it a 9/10, and was associated with nausea and three episodes of non-bloody, non-bilious emesis. Moving seemed to make the pain worse and nothing made it better. He denied any fever, chills, changes in bowel movement. He also denied any previous similar episodes in the past. The pain became progressively worse through the days which is what made him come into the hospital. On admission, his vitals were stable and labs were remarkable for leukocytosis and elevated pancreatic enzymes (Table 1).

**Table 1:** Laboratory values upon admission

	Values	Normal Range
White blood cell count	29.6	(4.8-10.8)
Lipase	522	(8-78)
Calcium	9.3	(8.4-10.2)
AST (Aspartate Aminotransferase)	19	(5-34)
ALT (Alanine Transaminase)	26	(11-55)
Total Bilirubin	1.0	(0.1-1.3)
Direct Bilirubin	0.5	(0.0-0.4)
Alkaline Phosphatase	108	(38-126)
Cholesterol	120	(130-200)
Triglycerides	78	(10-150)
LDL (low-density lipoprotein)	67	(83-210)
COVID-19 Antigen	Negative	Negative

**Figure 1:**
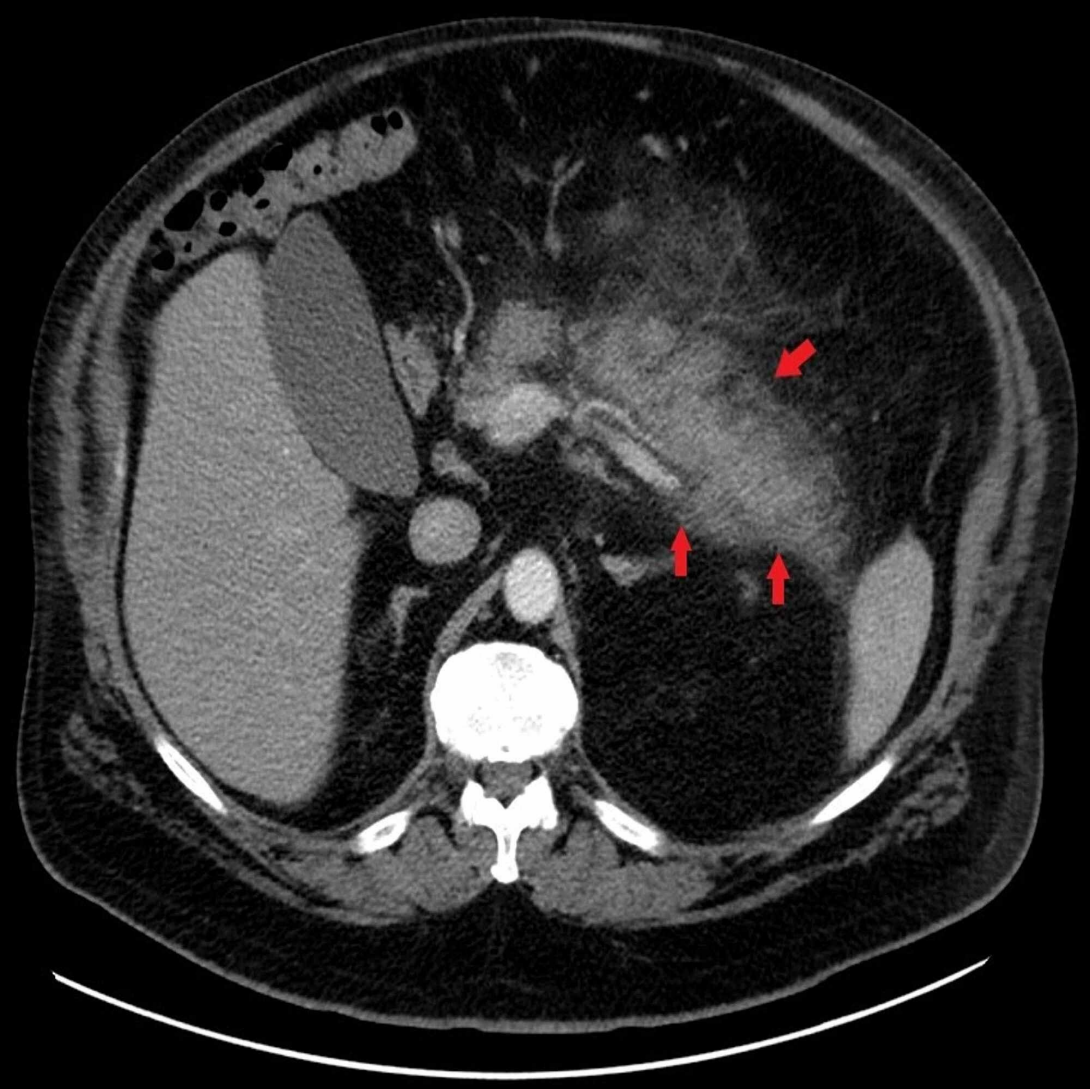
CT abdomen without contrast demonstrating diffuse peripancreatic stranding

An ultrasound of the abdomen was performed which showed a normal gallbladder without cholelithiasis and a lack of evaluation of the pancreas secondary to obscuration by overlying bowel gas. A computed tomography (CT) of the abdomen without contrast was then obtained which demonstrated diffuse peri-pancreatic stranding suggestive of acute pancreatitis (Figure 1).

He was subsequently made NPO, started on normal saline at 125 ml/hr, and given morphine 2 mg PRN Q4. After ruling out the main causes of pancreatitis and upon chart review of his medications, it was found that his meloxicam dose had just been doubled from 7.5 mg to 15 mg two weeks prior to presentation. Meloxicam was withheld during his hospital stay and discontinued upon discharge. Of note, the patient did spike a fever two nights in a row with some increase in his pain, both of which resolved on their own. But due to these events, a follow-up CT of the abdomen and pelvis was conducted which ruled out any complications such as fluid collection or pancreatic necrosis. The patient remained stable and was able to go home six days after admission. 

## Discussion

Within the field of gastroenterology, pancreatitis continues to maintain its position as one of the most common causes of hospitalizations secondary to a gastrointestinal disturbance in the developed world [1]. Pancreatitis is represented as inflammation of the pancreas through a specific path of physiologic mechanisms that cause the destruction of pancreatic acinar cells. Pancreatic acinar cell destruction may cascade into further inflammation secondary to activation of pro-inflammatory cell lines including granulocytes and macrophages. The physiology of pro-inflammatory cytokines depends on the acuity of pancreatitis. Pancreatitis typically is characterized as either chronic, acute, or acute on chronic [2]. In regards to this particular presentation, drug-induced pancreatitis usually presents as acute pancreatitis. In order to diagnose pancreatitis, several criteria must be met including two or more of the following: abdominal pain consistent with acute pancreatitis; this typically includes abdominal pain with radiation toward the back. The second criteria includes serum amylase and/or lipase which must be greater than three times the normal upper limit, the third criteria are suggestive findings seen on abdominal imaging such as pancreatic inflammation on abdominal CT scan.

Within the United States, the two most prevalent etiologies of acute pancreatitis are secondary to gallstones (which represented 35%-40% of cases), as well as ethanol (EtOH) use which represents approximately 30% of cases. Although these two factors account for the majority of cases, there is a plethora of other etiology such as hypertriglyceridemia, autoimmune pancreatitis, and drug-induced mechanism systems which may contribute to acute pancreatitis [3]. This particular case presentation represents acute pancreatitis secondary to a drug-induced mechanism. In order to diagnose drug-induced pancreatitis, the clinician must rule out all other etiologies which may contribute to pancreatitis including the mentioned gallstones, elevated triglycerides, and alcohol abuse. Then, a thorough review of the patient's lifestyle and medications must be achieved to observe for any particular inciting factors. This particular patient was noted to be taking meloxicam which was recently increased in dosage two weeks prior to presentation. This suspicious increase of the dosage of this nonsteroidal anti-inflammatory drug (NSAID) in combination with the lack of other known inciting etiologies which may contribute to pancreatitis helped determine that a drug-induced mechanism was likely the etiology. Although the mechanism to explain why NSAIDs may be implicated in acute pancreatitis has not been thoroughly confirmed, there has been speculation that NSAIDs may cause a lack of appropriate response directed against oxidative stress secondary to a reduction in systemic glutathione that results from decreased superoxide dismutase activity, as well as a possible destabilization effect of the NSAID on the pancreatic cell membrane secondary to their effects on prostaglandins [4]. A direct hypersensitivity and/or immune-mediated response directly from the NSAID in a particular individual who developed drug-induced pancreatitis cannot be completely ruled out [5].

The best treatment approach in these particular individuals is early identification of the pancreatitis and prompt discontinuation of the offending agent in order to mitigate the deleterious effects of continuous pancreatic inflammation [6]. Typically, drug-induced pancreatitis improves upon cessation of the offending agent, this leads to decreased morbidity and mortality from advanced pancreatitis including the possibility of developing a pancreatic pseudocyst, chronic pancreatitis as well necrotizing pancreatitis [7]. For these reasons, it is crucial to keep a high index of clinical suspicion for drug-induced etiologies when evaluating a patient with pancreatitis in whom all other common scenarios have been ruled out.

## Conclusions

Though not uncommon, we are starting to see more and more cases of drug-induced pancreatitis across the world. Although physicians might not think about such an etiology right away, it is important to keep it in mind and do a thorough review of the patient's medications. We present this case in order to bring attention to an important etiology that might not be very obvious at first.
